# Evidence for oxygenation of Fe-Mg oxides at mid-mantle conditions and the rise of deep oxygen

**DOI:** 10.1093/nsr/nwaa096

**Published:** 2020-05-09

**Authors:** Jin Liu, Chenxu Wang, Chaojia Lv, Xiaowan Su, Yijin Liu, Ruilian Tang, Jiuhua Chen, Qingyang Hu, Ho-Kwang Mao, Wendy L Mao

**Affiliations:** Center for High Pressure Science and Technology Advanced Research (HPSTAR), Beijing 100094, China; Department of Geological Sciences, Stanford University, Stanford, CA 94305, USA; Department of Geological Sciences, Stanford University, Stanford, CA 94305, USA; Center for High Pressure Science and Technology Advanced Research (HPSTAR), Beijing 100094, China; School of Earth and Space Sciences, Peking University, Beijing 100871, China; SLAC National Accelerator Laboratory, Menlo Park, CA 94025, USA; Center for High Pressure Science and Technology Advanced Research (HPSTAR), Beijing 100094, China; Center for Study of Matter at Extreme Conditions, Department of Mechanical and Materials Engineering, Florida International University, Miami, FL 33199, USA; Center for High Pressure Science and Technology Advanced Research (HPSTAR), Beijing 100094, China; Center for High Pressure Science and Technology Advanced Research (HPSTAR), Beijing 100094, China; Department of Geological Sciences, Stanford University, Stanford, CA 94305, USA; SLAC National Accelerator Laboratory, Menlo Park, CA 94025, USA

**Keywords:** subducting slab, hydrous phase, lower mantle, deep oxygen, oxygen-excess oxides

## Abstract

As the reaction product of subducted water and the iron core, FeO_2_ with more oxygen than hematite (Fe_2_O_3_) has been recently recognized as an important component in the D” layer just above the Earth's core-mantle boundary. Here, we report a new oxygen-excess phase (Mg, Fe)_2_O_3+_*_δ_* (0 < *δ* < 1, denoted as ‘OE-phase’). It forms at pressures greater than 40 gigapascal when (Mg, Fe)-bearing hydrous materials are heated over 1500 kelvin. The OE-phase is fully recoverable to ambient conditions for *ex situ* investigation using transmission electron microscopy, which indicates that the OE-phase contains ferric iron (Fe^3+^) as in Fe_2_O_3_ but holds excess oxygen through interactions between oxygen atoms. The new OE-phase provides strong evidence that H_2_O has extraordinary oxidation power at high pressure. Unlike the formation of pyrite-type FeO_2_H*x* which usually requires saturated water, the OE-phase can be formed with under-saturated water at mid-mantle conditions, and is expected to be more ubiquitous at depths greater than 1000 km in the Earth's mantle. The emergence of oxygen-excess reservoirs out of primordial or subducted (Mg, Fe)-bearing hydrous materials may revise our view on the deep-mantle redox chemistry.

## INTRODUCTION

Iron and oxygen are the most abundant elements on Earth by mass and number of atoms, respectively. Their compounds serve as important controls on the redox of our planet. Extensive research efforts have been devoted to the synthesis and characterization of a large number of iron oxides [1–6]. However, the composition range has long stayed between Fe and Fe_2_O_3_ (i.e. O/Fe ratios of 0–1.5), until the discovery of the pyrite-structured FeO_2_ (denoted as ‘Py-phase’) when water meets iron near the core-mantle boundary [6–10]. The reaction has a number of important consequences such as the formation of ultra-low sound velocity zones at the D” layer [7] and an internal source for the Great Oxidation Event [8]. Moreover, this opens a new important area of oxides with a O/Fe ratio above 1.5.

Here we searched for iron oxides between Fe_2_O_3_ and FeO_2_ (O/Fe = 1.5–2.0) and discovered an oxygen-excess phase, (Mg,Fe)_2_O_3_**_+_***_δ_* (0 < *δ* <1, denoted as ‘OE-phase’) with extra oxygen compared to ferropericlase (Mg,Fe)O and hematite (Fe_2_O_3_). The OE-phase emerged when iron-rich hydrous materials were exposed to laser heating at pressures greater than 40 gigapascal. This phase could coexist with the Py-phase at deep mantle conditions, whereas the two phases are distinct in crystal chemistry. The OE-phase can be pressure quenched to ambient conditions as a metastable phase, and thus is ready for further chemical analysis using transmission electron microscopy (TEM). The results indicate that iron is in the ferric state (Fe^3+^) while the valence of oxygen is greater than −2 in conventional oxides. Furthermore, we discovered the OE-phase can incorporate a significant amount of Mg to generate (Mg, Fe)_2_O_3_**_+_***_δ_* at mid-mantle conditions. The OE-phase may represent a new structural prototype that may be tolerable to substitution by other similar cations (e.g. Al, Ti, Ni and Cu) as well as accommodation for volatiles (e.g. C, noble gases and halogens), leading to a large family of new compounds at high *P-T* conditions. The peculiar oxygen chemistry in the OE-phase may drastically revise our view of crystal chemistry in the deep interior of Earth and other planets.

## RESULTS

We conducted a series of *in situ* high *P-T* synchrotron X-ray diffraction (XRD) measurements on iron-rich hydrous materials up to the lowermost mantle *P-T* conditions using laser-heated diamond-anvil cell (LHDAC) techniques at beamlines 13ID-D and 16ID-B of the Advanced Photon Source, Argonne National Laboratory, USA and at BL10XU of SPring-8, Japan [11]. Starting materials include ferropericlase (Mg,Fe)O mixed with brucite Mg(OH)_2_, and goethite FeOOH mixed with hematite Fe_2_O_3_ with stoichiometric O/Fe ratios of 1.5–2.0, with the understanding that goethite would lose its hydrogen when being heated to sufficiently high temperature (>1500–2000 K) [6]. The three mixtures of goethite and hematite with O/Fe ratios of 1.67, 1.75 and 1.83 were mechanically ground for one hour, respectively, in a glove box filled with Ar. To prevent adsorption of O_2_, H_2_O or CO_2_, the sample assemblage was evacuated for a half hour before being sealed in vacuum using a high-pressure gas loading system. Ne, Ar and LiF were used as oxygen-free and iron-free pressure-transmitting medium and thermal insulation.

**Figure 1.**
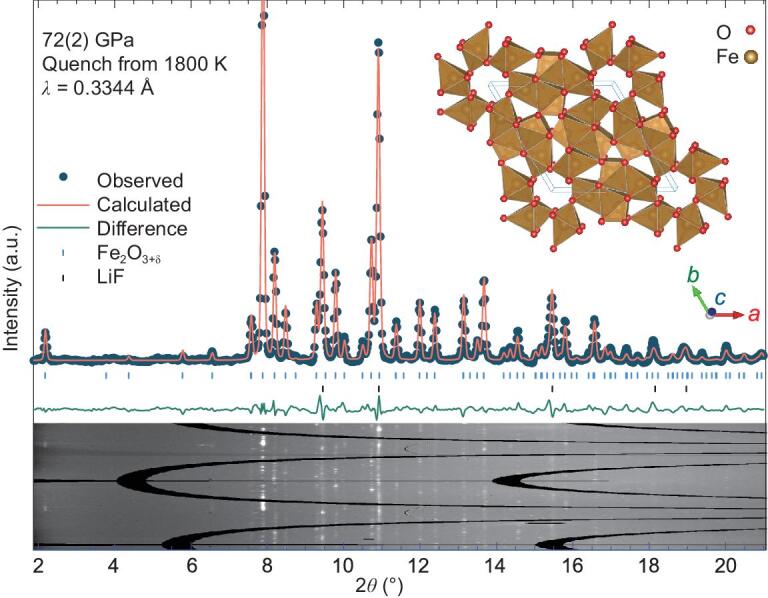
X-ray diffraction pattern collected at 72(2) GPa and room temperature. The assemblage was compressed to the target pressure at room temperature and then laser heated to 1800 K. The pure OE-phase synthesized was from the mixture of goethite (FeOOH) and hematite (Fe_2_O_3_) with an O/Fe ratio of 1.75, corresponding to Supplementary Fig. 1B. X-ray wavelength was 0.3344 Å. It was fitted by Rietveld refinement to the OE-phase Fe_2_O_3+δ_ and LiF. The *R*-factor is *wR_f_* = 0.113. Inset: Fe_12_O_18_ framework with excess oxygen (δ) to fill up the channel space at each corner of the unit cell.

Upon laser heating, we consistently observed a set of new diffraction lines as the dominant OE-phase that can be indexed to a large unit cell with *a* = *b*, *α* = *β* = 90° and *γ* = 120°, together with the previously known phases of Fe_2_O_3_, FeOOH and pressure medium. We note that the OE-phase which formed at high *T* was stable at room *T* at high *P*. In general, the XRD pattern collected from the mixture with an O/Fe ratio of 1.67 showed unreacted residual Fe_2_O_3_ (Supplementary Fig. 1A), while the XRD pattern from that of 1.83 showed residual FeOOH (Supplementary Fig. 1C). A pure OE-phase without other iron oxides was synthesized from the mixture with the O/Fe ratio of 1.75 at 72(2) GPa and 1800 K (Fig. 1 and Supplementary Fig. 1B). Considering the composition uncertainty of these mixtures, the O/Fe ratio of the OE-phase is likely between 1.67 (Fe_2_O_3.33_) and 1.83 (Fe_2_O_3.67_) with the *δ* value ranging from 0.33 to 0.67 in Fe_2_O_3_**_+_***_δ_*. The OE-phase was indexed to a unit cell of *a* = *b* = 10.100(1) Å, *c* = 2.634(1) Å, *α* = *β* = 90°, *γ* = 120° and *V* = 232.7(1) Å^3^, with 1σ uncertainties in parenthesis at 72(2) GPa (Table 1). Moreover, the non-overlapping spotty XRD pattern of the new phase makes it suitable for multigrain crystallographic analysis [12–14]. We applied the multigrain method to XRD experiments on goethite at 91(3) GPa and 1600–2200 K with Ar as the pressure-transmitting medium (Supplementary Fig. 2). The orientation matrices of two crystallites were identified and each had over one hundred unique reflections corresponding to three variables of rotation (*ω*), azimuth (*η*), and Bragg (2*θ*) angles (Supplementary Tables 1 and 2). The two single-crystal datasets provide robust confirmation of the unit cell assignment.

**Table 1.** Powder XRD pattern for the OE-phase at 72(2) GPa at room temperature. The OE-phase was synthesized from the starting mixture of goethite (FeOOH) and hematite (Fe_2_O_3_) with an O/Fe ratio of 1.75 and has lattice parameters of *a* = 10.100(1) Å, *c* = 2.634(1) Å and *V* = 232.7(1) Å^3^ (Fig. 1). X-ray wavelength is 0.3344 Å.

	*d*-obs	2*θ*-obs	2*θ*-calc	2*θ*-diff	Int.
*h k l*	(Å)	(°)	(°)	(°)	(a.u.)
1 0 0	8.7333	2.194	2.191	0.003	18
2 1 0	3.3065	5.797	5.798	−0.001	3
3 0 0	2.9178	6.570	6.575	−0.005	5
1 0 1	2.5235	7.598	7.602	−0.004	50
3 1 0	2.4263	7.903	7.904	−0.001	100
1 1 1	2.3357	8.210	8.211	−0.001	85
2 0 1	2.2564	8.499	8.499	0.000	40
2 1 1	2.0596	9.313	9.310	0.003	30
3 0 1	1.9539	9.818	9.814	0.004	70
2 2 1	1.8230	10.525	10.526	−0.001	15
3 1 1	1.7846	10.752	10.753	−0.001	65
4 0 1	1.6824	11.407	11.406	0.001	30
3 2 1	1.5965	12.023	12.025	−0.002	25
4 1 1	1.5459	12.418	12.420	−0.002	30
6 0 0	1.4574	13.175	13.172	0.003	40
3 3 1	1.4182	13.541	13.539	0.002	30
5 2 0	1.4009	13.709	13.712	−0.003	50
5 1 1	1.3495	14.234	14.237	−0.003	10
6 1 0	1.3342	14.398	14.402	−0.004	10
0 0 2	1.3167	14.591	14.587	0.004	30
6 0 1	1.2747	15.074	15.064	0.010	10
4 4 0	1.2624	15.222	15.221	0.001	10
2 2 2	1.1678	16.463	16.464	−0.001	5

**Figure 2.**
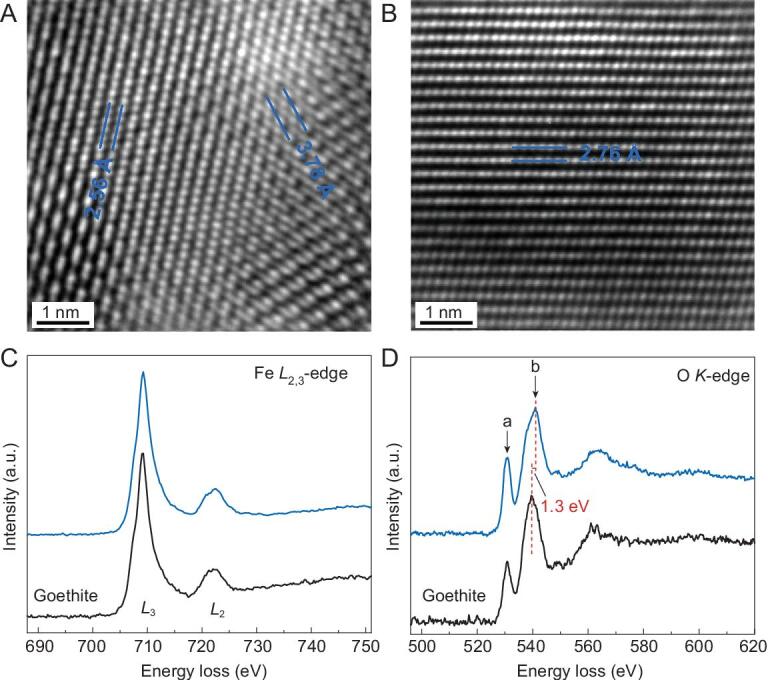
High-resolution TEM images and EELS spectra observed from two different grains (A and B) in the recoverable Fe_2_O_3+δ_ sample. The *d*-spacings between the blue lines in (A) are 3.78(1) Å and 2.56(1) Å, corresponding to (120) and (021) planes, respectively. The one in (B) is 2.76(1) Å, corresponding to (130) plane. (C) The Fe *L*_2,3_-edges and (D) O *K*-edges of the OE-phase (blue) and goethite (black), respectively, after background subtraction.

Unlike most other unquenchable high-pressure phases (e.g. the Py-phase and post-perovskite silicates), the OE-phase was recoverable back to ambient conditions from decompression at room temperature (Supplementary Figs 3 and 4). The recovered samples were further prepared using focused ion beam (FIB) and analyzed using TEM to investigate their composition and chemical state (Fig. 2 and Supplementary Figs 5 and 6). The high-resolution TEM images of a quenched sample in Fig. 2 showed that *d*-spacings of 3.78(1), 2.76(1) and 2.56(1) Å were observed, consistent with (120), (130) and (021) planes of the OE-phase at ambient conditions. The three *d*-spacing values observed from different orientations do not belong to either hematite or goethite, indicating the distinguishable structure of the OE-phase. Meanwhile, TEM energy-dispersive X-ray spectroscopy (EDS) measurements showed the O/Fe ratios of hematite (Fe_2_O_3_), the OE-phase and goethite (FeOOH) to be 1.47(4), 1.80(6) and 2.12(6), respectively, with 1σ uncertainties in parenthesis (Supplementary Fig. 6 and Supplementary Table 3). That is, the O/Fe ratio of the OE-phase is between Fe_2_O_3_ and FeOOH, consistent with our XRD experiments.

The electron energy loss spectra (EELS) of Fe *L*_2,3_-edge and O *K*-edge near-edge structures were collected for the OE-phase and goethite. The Fe *L*_3_-edge onset of the OE-phase is the same as goethite at 709.3 eV, indicating no chemical shift and iron in the ferric state (Fig. 2C). On the other hand, the intensities of the O *K*-edge pre-peak at 531.0 eV (labeled as *a* in Fig. 2D) differ between the OE-phase and goethite, attributable to distinct oxygen bonding environments and symmetry variation [15]. Notably, the O *K*-edge peak position of the OE-phase is at 541.0 eV, higher than that of FeOOH by ∼1.3 eV (labeled as *b* in Fig. 2D), further demonstrating that oxygen bonding environments in the OE-phase are distinct from goethite with hydroxyl groups (OH). This is in agreement with hydroxyl or hydrogen-related bonds not visible between 3000–4000 cm^−1^ in laser Raman spectroscopic measurements on the OE-phase (Supplementary Fig. 7). The absence of sharp OH vibration modes suggested that little hydrogen might be structurally bonded in the ferric OE-phase. In addition, the molar volume for the OE-phase, *V* = 221.3(2) Å^3^ at 91(3) GPa, is smaller than the sum of the volumes for Fe_2_O_3_ ([4]) and 2FeO_2_ ([16]), *V* = 221.9(7) Å^3^. That is, the OE-phase might be nominally anhydrous. Hence, the effective valence of oxygen would be roughly between −1.6 and −1.8 in the OE-phase, distinct from oxygen anions O^2−^ in common oxides Fe_2_O_3_ and FeO.

To further assess the crystal packing and stoichiometry of the OE-phase, the Rietveld full-profile refinement method was applied to the powder X-ray diffraction data of the OE-phase at 72(2) GPa (Fig. 1). The atomic structure of Fe in the OE-phase was reasonably determined with the Fe_12_O_18_ framework in a unit cell in the *P*6_3_ (#173) symmetry (inset to Fig. 1 and Supplementary Table 4). Moreover, the OE-phase likely stores a varying amount of excess oxygen or little hydrogen filling up the channel space at each corner of the unit cell (inset to Fig. 1). We note that, in comparison to Fe atoms, O atoms slightly interact with X-rays and only contribute ∼10% of diffraction signals of the OE-Phase. Oxygen atomic positions were thus not well constrained with large uncertainties and should be used with caution (Supplementary Table 4). Evaluation of oxygen positions is beyond the scope of this study and definitely needs further investigation.

## IMPLICATIONS

**Figure 3.**
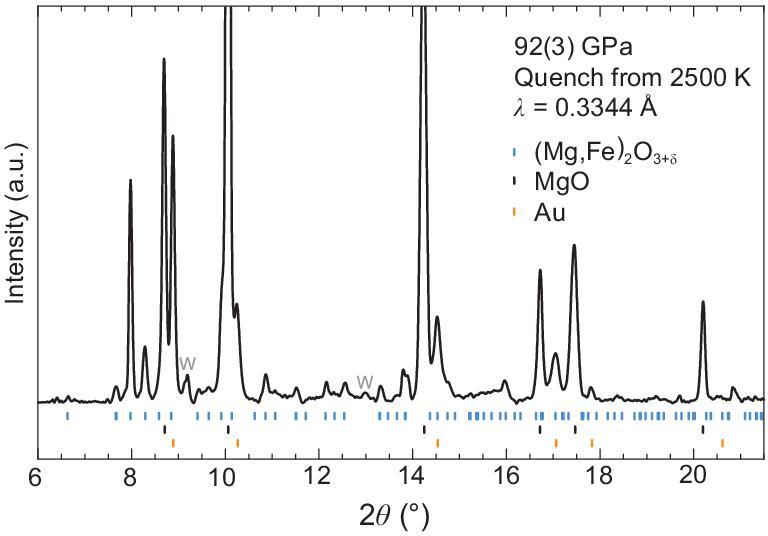
Representative X-ray diffraction pattern of the Mg-rich OE-phase at 92 GPa and room temperature. Starting materials were the mixture of ferropericlase (Mg_0.6_Fe_0.4_)O and brucite Mg(OH)_2_ and they reacted to form the Mg-rich OE-phase (*a* = 10.01(7) Å, *c* = 2.607(3) Å) upon laser heating at 92 GPa and 2 500(200) K. Au (*a* = 3.739 Å) was used as the pressure calibrant and W represents the tungsten gasket.

The crystal structure of the OE-phase may represent a structure prototype and accommodate other Earth-abundant components (e.g. Mg, Al, Ca, Ti and Ni). To evaluate whether the ferric OE-phase can incorporate Mg, we carried out laser-heated XRD experiments on a mixture of ferropericlase (Mg_0.6_Fe_0.4_)O and brucite Mg(OH)_2_ and observed the formation of the Mg-bearing OE-phase, (Mg, Fe)_2_O_3+δ_ under the *P-T* conditions of the mid-mantle (Fig. 3). Mg substitution will generally stabilize the phase to greater *P-T* conditions. In addition, the channel space in the OE-phase may offer a great flexibility not only for excess oxygen, but also for other volatiles (e.g. N, S, F and Cl). Considering its structural versatility, the OE-phase could be an important volatile carrier in the deep mantle.

The widespread presence of the OE-phase makes it and other oxygen-enriched oxides an important subject for the full range of future geochemistry and mineral physics studies [17,18]. The rich redox chemistry of the Earth was thought to be controlled by a series of iron oxides from FeO, Fe_5_O_6_, Fe_4_O_5_, Fe_7_O_9_, Fe_3_O_4_, Fe_5_O_7_, Fe_13_O_19_, to Fe_2_O_3_, that cover the conventional range with O/Fe ratios between 1.0 and 1.5 [1–5]. Now we have found that for the deep mantle the range may double to include FeO_2_ and the OE-phase. Meanwhile, recent global seismological observations [19,20] revealed a number of kilometer-scale S-to-P scattering objects in the mid-mantle at depths from ∼1000 to ∼1800 km, for instance, beneath the circum-Pacific area. Meanwhile, the OE-phase emerges at depths greater than 1000 km, suggesting that the distinct oxygen-excess reservoirs may contribute to mid-mantle seismic scatterings. Furthermore, as the formation of the OE-phase requires less hydrous materials, it may be more ubiquitous than FeO_2_, making the (Mg,Fe)-bearing OE-phase a promising candidate for explaining the unusual seismic features in the lower mantle.

**Figure 4.**
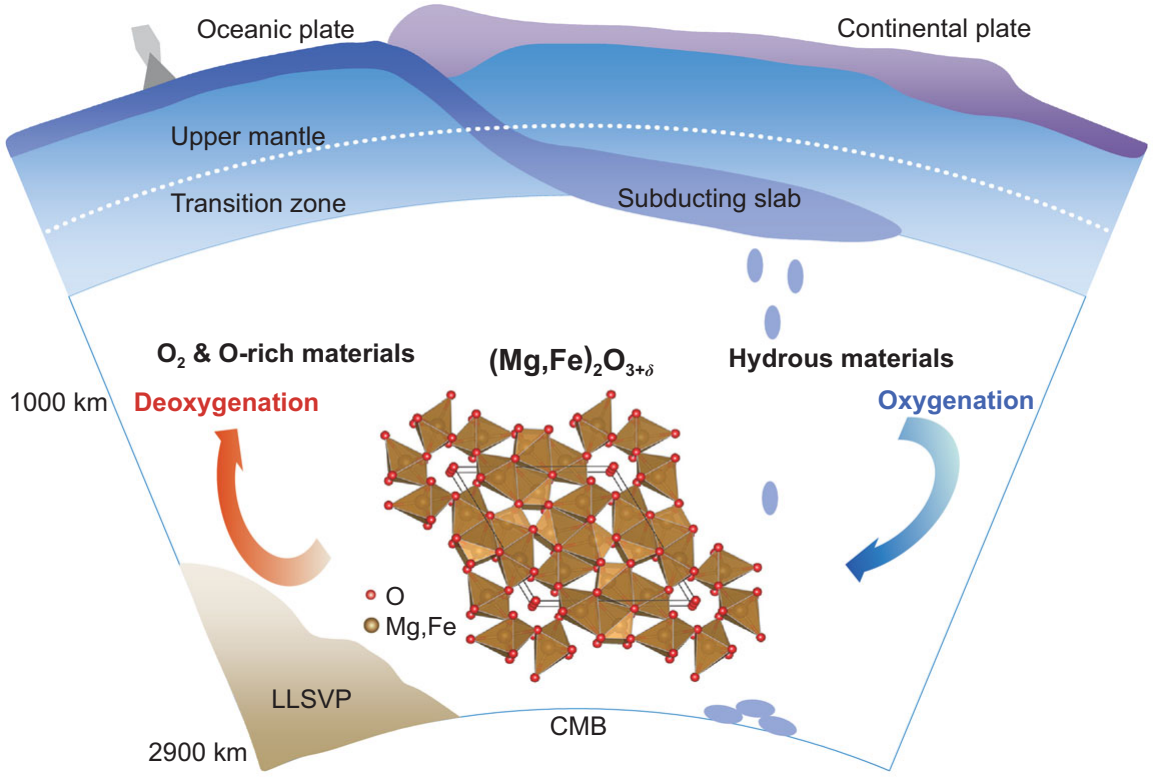
Schematic diagram of the deep oxygen factory inside the Earth.

The OE-phase can accommodate >10% more oxygen than ferropericlase (Mg,Fe)O and the most oxidized form of iron (Fe_2_O_3_) on Earth's surface. Through the peculiar chemistry of deep-mantle oxygen, it is conceivable that mantle components can store more oxygen than previously thought [21]. In Earth's early history, the primordial water [22] in the deep mantle would constructively build up oxygen-excess reservoirs containing the OE-phase. After plate tectonics started approximately three billion years ago [23], oxygen distribution would become more inhomogeneous within the hydrated mantle of most slab remnants, considering the water flux of about 3 × 10^11^ kg yr^−1^ into the deep mantle [24,25]. Consequently, long-term water circulation would facilitate the accumulation of oxygen-excess reservoirs in a portion approximately 2/3 of the mantle at depths greater than 1000 km (Fig. 4). Mantle components in the region are estimated to hold excess oxygen >100 times the mass of atmospheric oxygen (O_2_). Together with excess Fe^3+^ from the primordial lower mantle after Earth's core-mantle segregation [26], those oxygen-excess materials may have oxidized the shallow mantle and crust in the long term. The ancient continental crust evolving from mafic to felsic allowed free oxygen to build up in the Earth's atmosphere [27], which is fundamental to the evolution and habitability of complex life.

## CONCLUSION

The rare case that the OE-phase is quenchable, and the robustness of the quenched sample to sustain electron bombardment in TEM studies, offer the opportunity for in-depth study of its crystal chemistry, as well as a challenge to search for it in nature as diamond inclusions or meteorite shock products. In fact, most compounds that are synthesized under lower mantle conditions and are quenchable back to ambient conditions have been discovered and named as minerals, as shown by the examples of bridgmanite [28] and seifertite [29]. The (Mg,Fe)-bearing OE-phase is likely to follow suit.

## METHODS

### Synchrotron X-ray diffraction experiments

As regards the XRD measurements, a highly monochromatized incident X-ray beam was used with an energy of 30.493 keV (0.4066 Å) for the former and 37.077 keV (0.3344 Å) for the latter. The incident X-ray beam was focused down to a beam size of 2–5 μm full width at half maximum (FWHM) at sample position. For laser heating XRD experiments, two infrared laser beams were focused down to 20–30 μm FWHM on both sides of the sample and they were co-axially aligned with the incident X-ray beam using the X-ray induced luminescence on the sample and/or ruby [30,31]. The temperature of the heated samples was calculated through fitting the measured thermal radiation spectra with the gray-body assumption and its uncertainty was within 100–200 K based on multiple temperature measurements on both sides. Pressure was determined from lattice parameters of Au, Ne and/or Pt [32] generally with an uncertainty of 1–3 GPa.

### Multigrain indexation

This method is an extremely powerful tool for processing XRD data of multiple crystals that coexist with multiple phases from high-pressure experiments. Multigrain indexation can be used to extract single-crystal lattice parameters for each crystallite grain with definitive separation and characterization of an unknown phase [14,16]. Multigrain X-ray diffraction experiments were implemented at HPCAT, Argonne National Laboratory. Diffraction patterns were collected in the same fashion as single-crystal crystallography on a charge-coupled device (CCD) detector with micro-focused X-ray beam (beam size of 10 μm by 8 μm) and beam energy of 30.493 keV. A total number of 80 images were taken at each angle from −19.5° to 20.5°, with a scanning step of 0.5°. We indexed two grains from the X-ray diffraction pattern (Supplementary Fig. 2 and Supplementary Tables 1 and 2).

### TEM analysis and EELS measurements

TEM cross-sectional samples were prepared using FEI Helios NanoLab 600i DualBeam FIB/SEM at the Stanford Nano Shared Facilities (SNSF). The quenched sample was milled at 30 kV Gallium ion beam and lifted out using the micro omniprobe, followed by final thinning down to obtain a wedge for sufficient electron transparency. TEM analyses were performed using a 200 kV Tecnai F20 transmission electron microscope with EDS with super ultra-thin window (SUTW) and analyzer. The high-resolution TEM images were filtered and processed using DigitalMicrograph software for a better analysis of the *d*-spacings. EELS data were collected using an aberration-corrected FEI Titan 80–300 environmental TEM equipped with a Quantum 966 EEL spectrometer operated at 300 kV. Dual EELS acquisition employed in this study allowed both zero-loss peak and core-losses to be acquired simultaneously, the former of which could be used for energy calibration.

## Supplementary Material

nwaa096_Supplemental_File
