# Supplementary material for: Evidence for oxygenation of Fe-Mg oxides at mid-mantle conditions and the rise of deep oxygen
Source: Natl Sci Rev. 2020 May 9;8(4):nwaa096. doi: 10.1093/nsr/nwaa096 (PMC8288346; doi:10.1093/nsr/nwaa096)
Supplement: nwaa096_Supplemental_File [file nwaa096_supplemental_file.docx]

**SUPPLEMENTARY DATA**

**Evidence for oxygenation of Fe-Mg oxides at mid-mantle conditions and the rise of deep oxygen**

Jin Liu^1,2^*†, Chenxu Wang^2^†, Chaojia Lv^1^, Xiaowan Su^3^, Yijin Liu^4^, Ruilian Tang^1^, Jiuhua Chen^5^, Qingyang Hu^1^*, Ho-Kwang Mao^1^, and Wendy L. Mao^2,4^

**Affiliations:**

^1^Center for High Pressure Science and Technology Advanced Research (HPSTAR), Beijing 100094, China.

^2^Department of Geological Sciences, Stanford University, Stanford, CA 94305, USA.

^3^School of Earth and Space Sciences, Peking University, Beijing 100871, China.

^4^SLAC National Accelerator Laboratory, Menlo Park, CA 94025, USA.

^5^Center for Study of Matter at Extreme Conditions, Department of Mechanical and Materials Engineering, Florida International University, Miami 33199, USA.


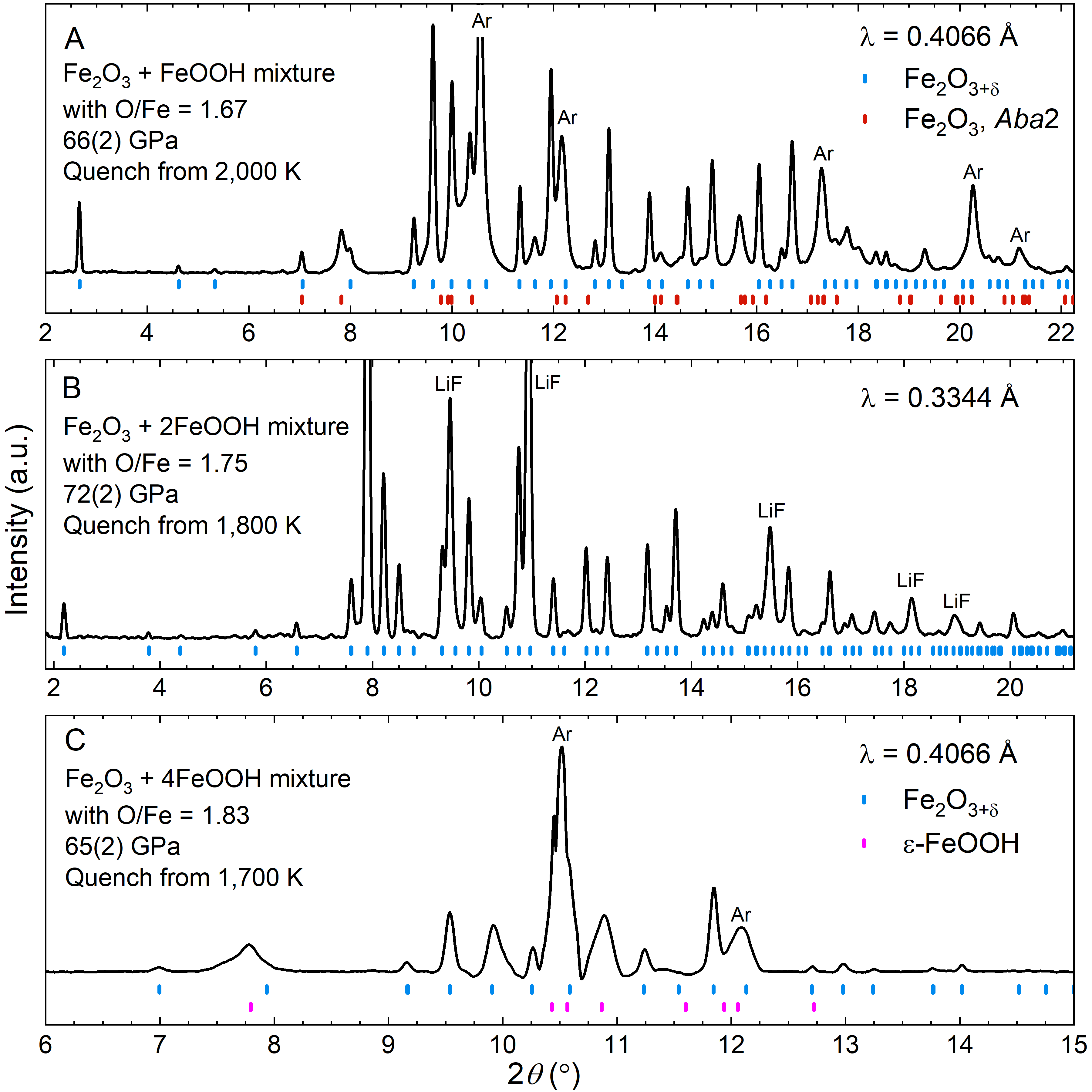


**Supplementary Figure 1.** Powder XRD patterns of the OE-phase Fe_2_O_3+δ_ synthesized from different mixtures of hematite (Fe_2_O_3_) and goethite (FeOOH) at high pressures and temperatures. Starting mixtures with an O/Fe ratio of 1.67 (***A***), 1.75 (***B***), and 1.83 (***C***), respectively. Ar or LiF were used as a pressure medium and thermal insulation.


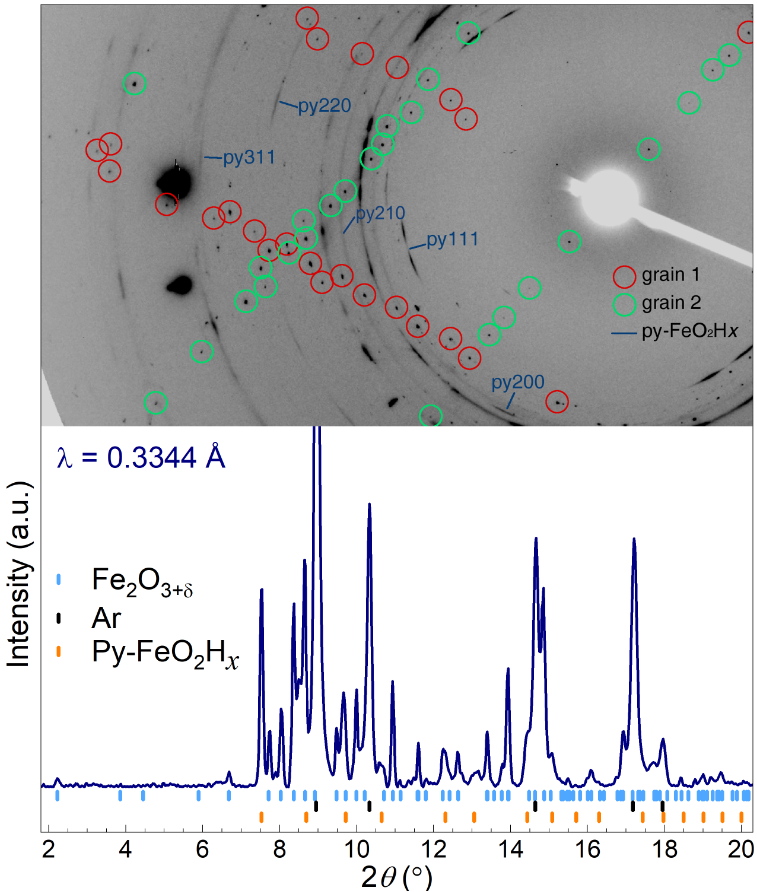


**Supplementary Figure 2.** Synthesis of the OE-phase in Ar at 91(3) GPa after temperature quench from 2,200 K. A section of the 2-dimensional XRD pattern is appended from the CCD detector. Circles in red and green are indexed for two crystallite grains of the OE-phase. Blue lines (py) indicate the Py-FeO_2_H*x* phase. Reflections and lattice parameters for the two crystallite grains #1 (marked by red circles) and #2 (marked by green circles) were listed in Supplementary Tables 1 and 2, respectively. The diamond anvils are responsible for the strongest single crystal diffraction spots.


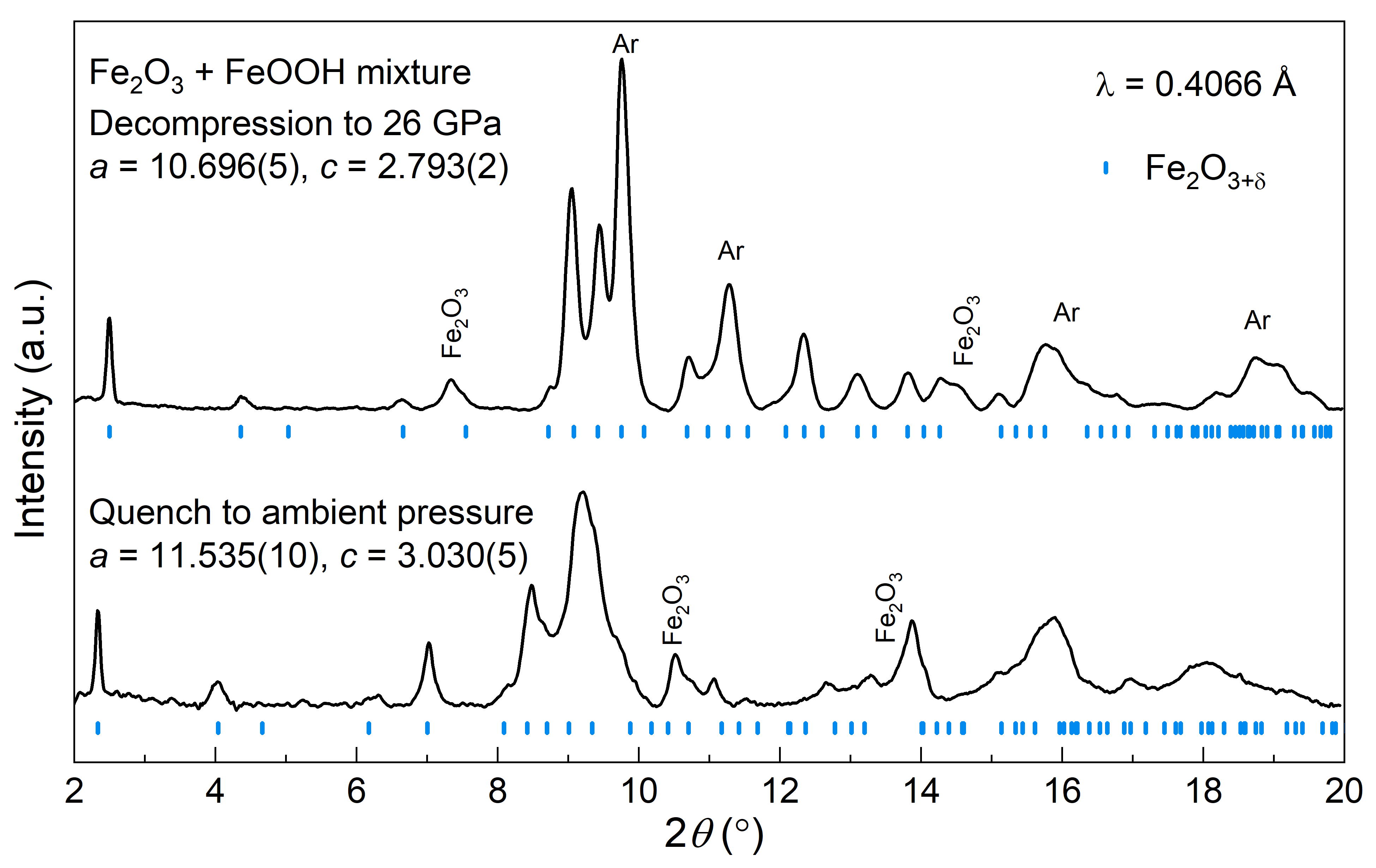


**Supplementary Figure 3.** Decompression of the OE-phase Fe_2_O_3+δ_ from 66 GPa to ambient pressure. The OE-phase was synthesized from the starting mixture of hematite (Fe_2_O_3_) and goethite (FeOOH) with an O/Fe ratio of 1.67 (please refer to Supplementary Fig. 1*A*). Ar was used as the pressure medium and thermal insulation. The OE-phase phase is recoverable back to ambient pressure and room temperature.


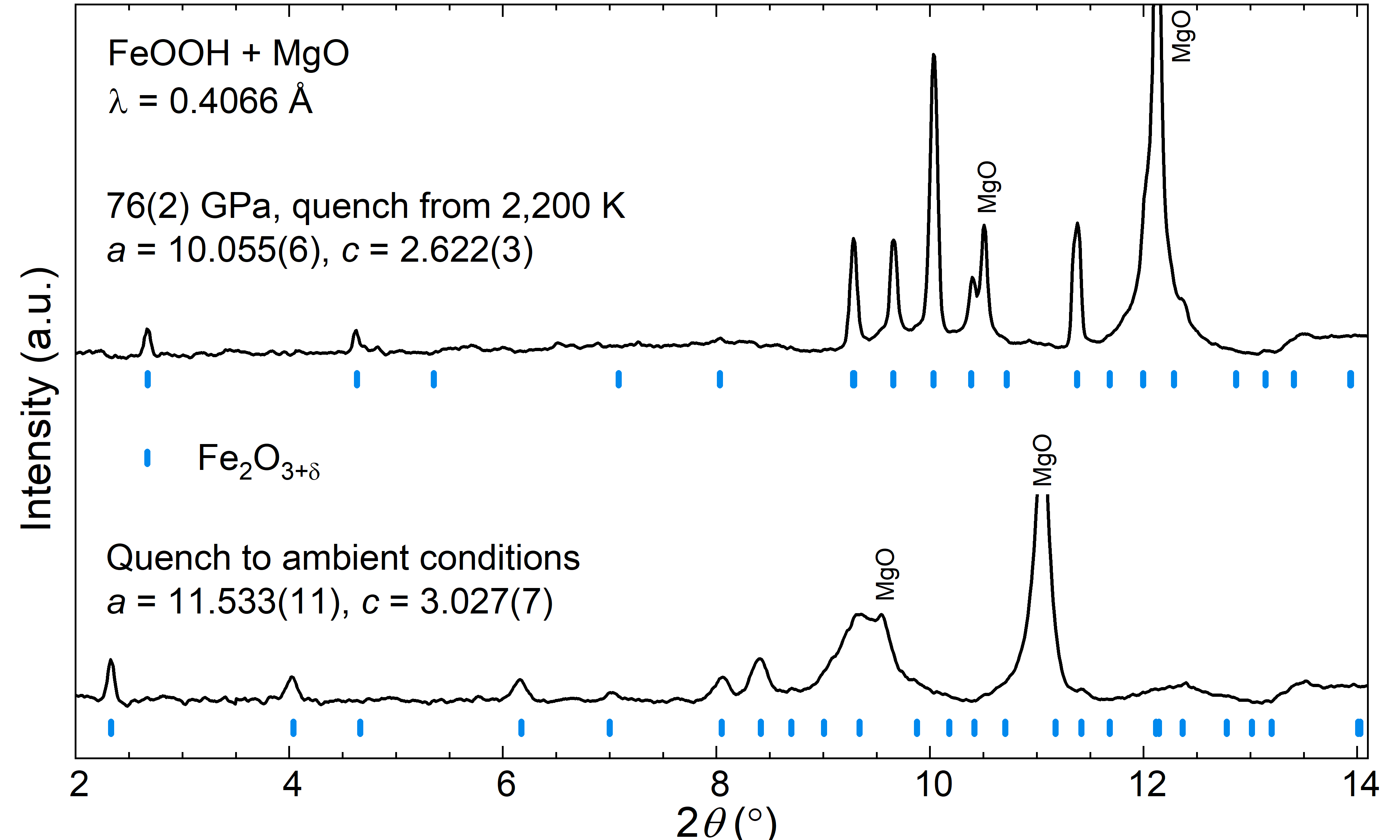


**Supplementary Figure 4.** The OE-phase Fe_2_O_3+δ_ recoverable to ambient pressure on cold decompression. The OE-phase synthesized from goethite (FeOOH) in the pressure-transmitting medium and thermal insulation of MgO.


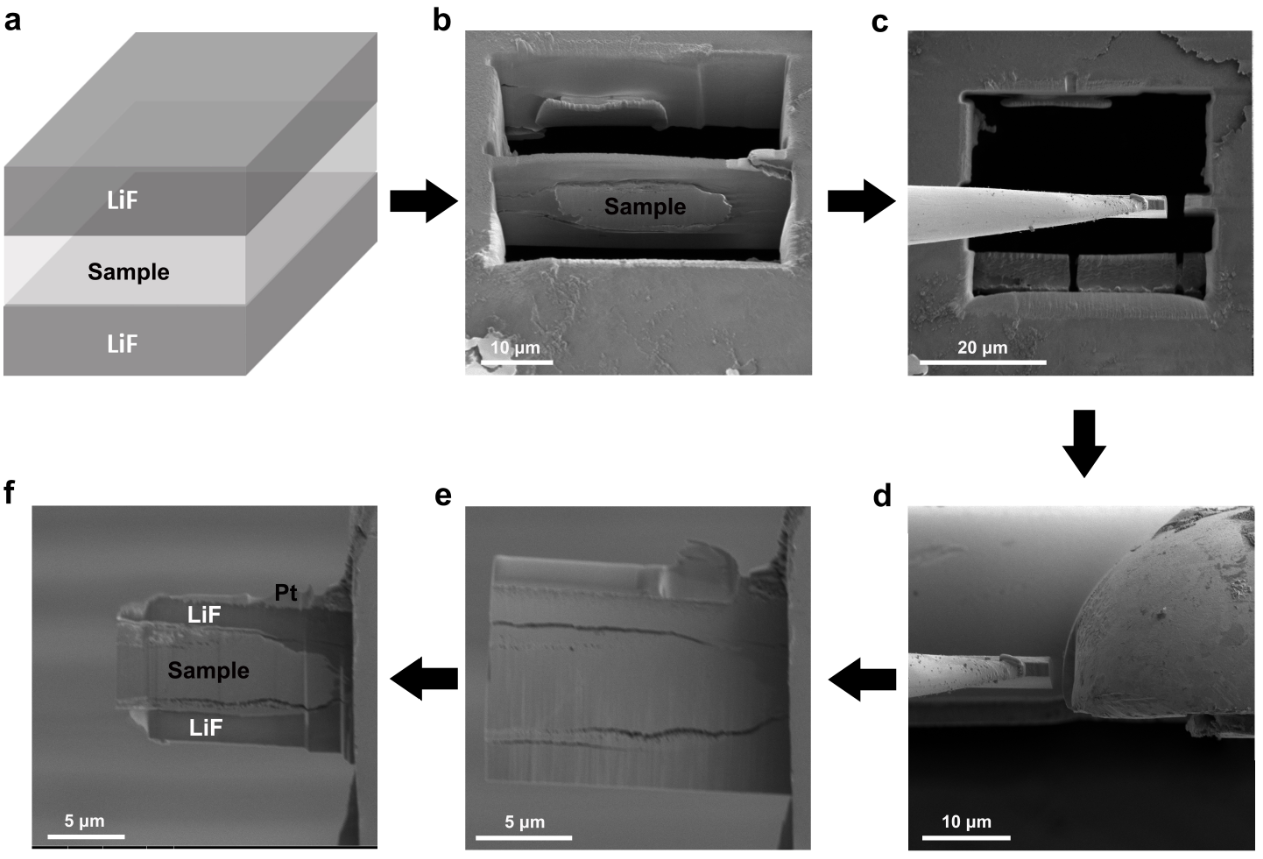


**Supplementary Figure 5.** The TEM sample preparation procedure for the quenched sample of the OE-phase Fe_2_O_3+δ_. (***a***) Model of the sandwich quenched sample in the DAC. (***b***) Sample trenches milled by Ga ion beam, with the cross section exhibiting the sample layer in between two thermal insulating layers. (***c***) and (***d***) Lift-out process of the sample using FIB. (***e***) SEM image of the sample adhered to the TEM grid. (***f***) Quenched sample after final thinning, which is thinner than 80 nm for sufficient electron transparency.


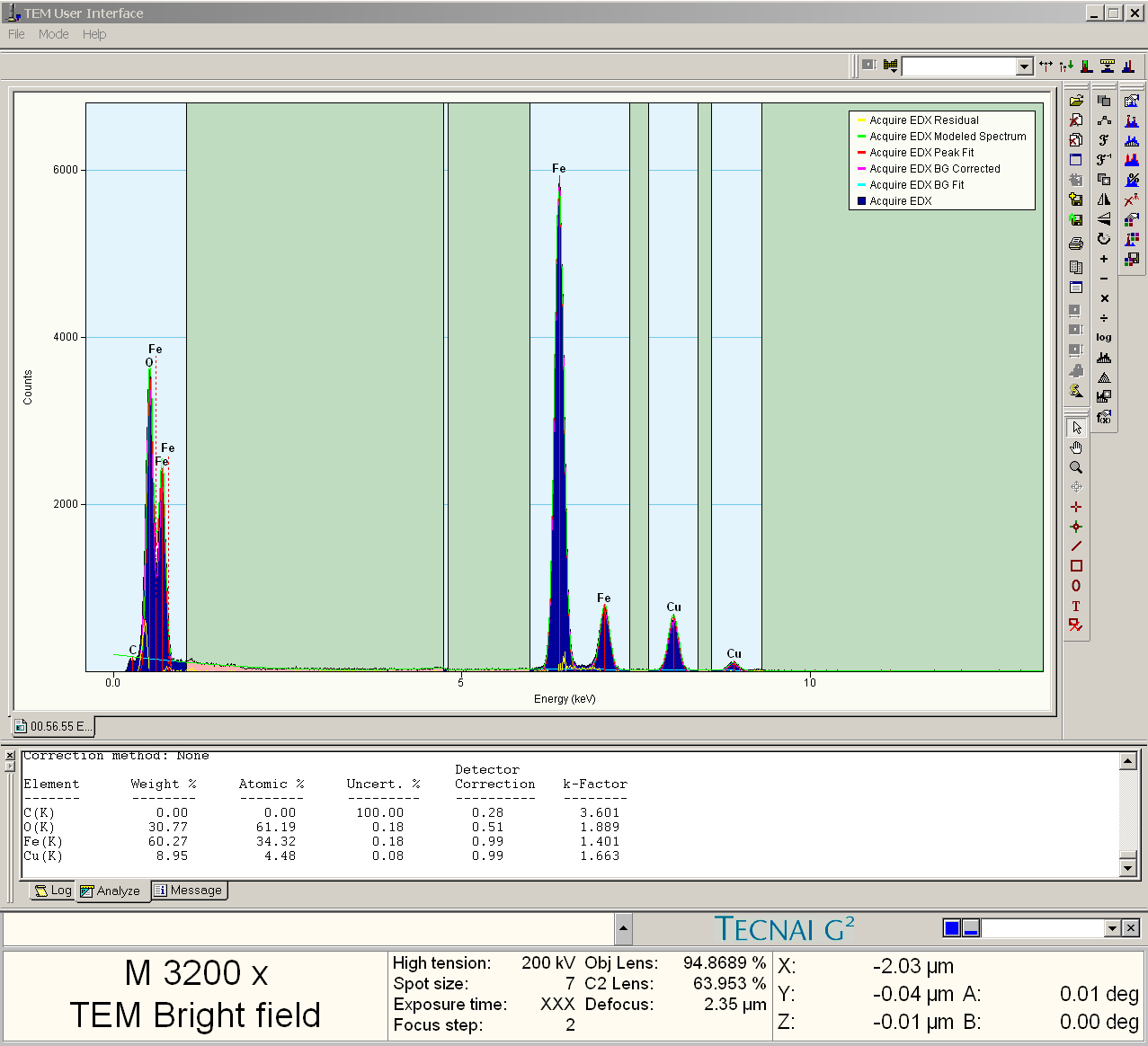


**Supplementary Figure 6.** TEM-EDS measurements on the recovered sample of the OE-phase. Cu signals were from the TEM copper grid holder.


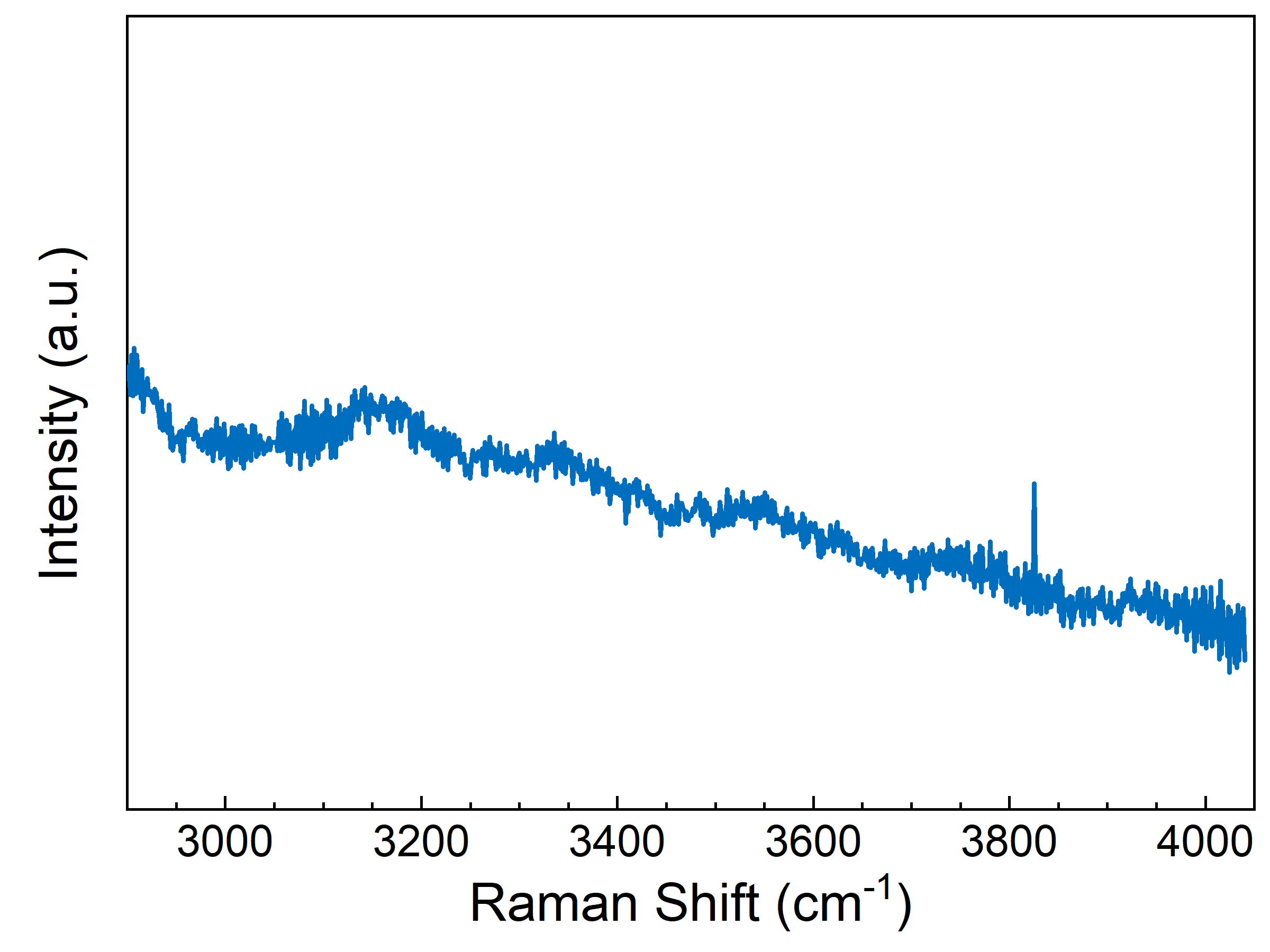


**Supplementary Figure 7.** Laser Raman spectra of the OE-phase at 70(2) GPa. The absence of sharp OH vibration modes between 3000 and 4000 cm^-1^ suggests that no hydrogen was structurally bonded in the ferric OE-phase.

**Supplementary Table 1.** X-ray diffraction reflections for the OE-phase. The reflections correspond to the crystallite grain #1 in Supplementary Fig. 2 (marked by red circles).

| *h k l* | 2*θ*_obs._ | 2*θ*_calc._ | *h k l* | 2*θ*_obs._ | 2*θ*_calc._ | *h k l* | 2*θ*_obs._ | 2*θ*_calc._ |
| --- | --- | --- | --- | --- | --- | --- | --- | --- |
| 0 -1  0 | 2.71 | 2.71 | 2 -5 -1 | 14.88 | 14.89 | -2 -1  2 | 19.49 | 19.49 |
| 0 -2  0 | 5.42 | 5.42 | 1 -6  0 | 15.10 | 15.11 | 1 -3 -2 | 19.49 | 19.49 |
| -1  3  0 | 7.15 | 7.17 | -1 -4  1 | 15.38 | 15.38 | -3  2  2 | 19.51 | 19.49 |
| 1 -3  0 | 7.16 | 7.17 | -1  5 -1 | 15.36 | 15.38 | -2  8  0 | 19.62 | 19.61 |
| 0 -3  0 | 8.14 | 8.13 | -1  5  1 | 15.40 | 15.38 | 2 -8  0 | 19.58 | 19.61 |
| 0  3  0 | 8.14 | 8.13 | 1 -5 -1 | 15.38 | 15.38 | -1 -6  1 | 20.01 | 20.01 |
| -1  0  1 | 9.43 | 9.42 | 0 -5 -1 | 16.32 | 16.32 | -1  7 -1 | 20.01 | 20.01 |
| -1  1  1 | 9.43 | 9.42 | 0 -5  1 | 16.30 | 16.32 | 1 -7 -1 | 20.01 | 20.01 |
| 0 -1 -1 | 9.41 | 9.42 | -3  7  0 | 16.50 | 16.52 | -2 -2  2 | 20.42 | 20.43 |
| 0  1 -1 | 9.40 | 9.42 | -3  6  1 | 16.77 | 16.77 | -2  4  2 | 20.43 | 20.43 |
| 1 -1 -1 | 9.41 | 9.42 | -2  7  0 | 16.95 | 16.97 | 2 -4 -2 | 20.44 | 20.43 |
| -1 -1  1 | 10.19 | 10.17 | 2 -7  0 | 16.93 | 16.97 | 1 -8  0 | 20.53 | 20.54 |
| -1  2  1 | 10.18 | 10.17 | -2  6 -1 | 16.95 | 16.99 | -3  4  2 | 20.62 | 20.61 |
| 1 -2 -1 | 10.17 | 10.17 | -2  6  1 | 17.00 | 16.99 | -1  4 -2 | 20.59 | 20.61 |
| -2  2  1 | 10.55 | 10.53 | 2 -6 -1 | 16.98 | 16.99 | -4  8  1 | 20.93 | 20.92 |
| 0 -2 -1 | 10.52 | 10.53 | -1 -5  1 | 17.63 | 17.63 | 0 -7  1 | 21.08 | 21.10 |
| 0 -4  0 | 10.84 | 10.84 | -1 -6  0 | 17.83 | 17.82 | -3  8  1 | 21.11 | 21.10 |
| -1  3  1 | 11.54 | 11.53 | -1  7  0 | 17.83 | 17.82 | 3 -8 -1 | 21.09 | 21.10 |
| 1 -3 -1 | 11.53 | 11.53 | 1 -7  0 | 17.80 | 17.82 | -2  8 -1 | 21.62 | 21.63 |
| -2  5  0 | 11.80 | 11.82 | 0  0 -2 | 18.08 | 18.10 | -2  8  1 | 21.65 | 21.63 |
| 0 -3 -1 | 12.15 | 12.16 | 0 -1 -2 | 18.29 | 18.31 | 2 -8 -1 | 21.62 | 21.63 |
| -1  5  0 | 12.42 | 12.43 | 0  1 -2 | 18.29 | 18.31 | -4  5  2 | 22.04 | 22.02 |
| 1 -5  0 | 12.42 | 12.43 | 1 -1 -2 | 18.31 | 18.31 | 1 -5 -2 | 22.03 | 22.02 |
| -2  4  1 | 13.04 | 13.03 | 1  0 -2 | 18.31 | 18.31 | -1 -7  1 | 22.47 | 22.48 |
| 2 -4 -1 | 13.03 | 13.03 | 0 -6 -1 | 18.67 | 18.66 | -1  8 -1 | 22.50 | 22.48 |
| -1  4  1 | 13.33 | 13.32 | 0 -6  1 | 18.64 | 18.66 | -1  8  1 | 22.51 | 22.48 |
| 1 -4 -1 | 13.31 | 13.32 | -1  2  2 | 18.70 | 18.71 | 1 -8 -1 | 22.47 | 22.48 |
| -1 -3  1 | 13.32 | 13.32 | 1 -2 -2 | 18.71 | 18.71 | -3  9  1 | 23.48 | 23.46 |
| -1  4 -1 | 13.28 | 13.32 | -2  1  2 | 18.72 | 18.71 | 3 -9 -1 | 23.44 | 23.46 |
| 0 -5  0 | 13.58 | 13.56 | 3 -7 -1 | 18.84 | 18.86 | -3  9 -1 | 23.44 | 23.46 |
| 0 -4 -1 | 14.12 | 14.12 | -2  0  2 | 18.92 | 18.91 | 1 -6 -2 | 23.66 | 23.67 |
| 0 -4  1 | 14.11 | 14.12 | 0  2 -2 | 18.90 | 18.91 | -2  9 -1 | 24.10 | 24.10 |
| -2  6  0 | 14.35 | 14.36 | 0 -7  0 | 19.03 | 19.03 | 2 -9 -1 | 24.09 | 24.10 |
| 2 -6  0 | 14.34 | 14.36 | -2  7 -1 | 19.22 | 19.25 | 0 -9  0 | 24.54 | 24.55 |
| -2  5  1 | 14.90 | 14.89 | -3  1  2 | 19.52 | 19.49 |  |  |  |

Data were collected at 91(3) GPa and room temperature. Observed and calculated 2-theta angles are compared in the table with incident beam wavelength *λ* = 0.4066 Å. The first crystallite grain has 104 reflections with lattice parameters of *a* = 9.942(1) Å and *c* = 2.5842(3) Å.

**Supplementary Table 2.** X-ray diffraction reflections for the OE-phase. The reflections correspond to the crystallite grain #2 in Supplementary Fig. 2 (marked by green circles).

| *h k l* | 2*θ*_obs._ | 2*θ*_calc._ | *h k l* | 2*θ*_obs._ | 2*θ*_calc._ | *h k l* | 2*θ*_obs._ | 2*θ*_calc._ |
| --- | --- | --- | --- | --- | --- | --- | --- | --- |
| 2 -2  0 | 5.40 | 5.42 | -6  5  0 | 15.10 | 15.11 | 7 -7  0 | 19.01 | 19.03 |
| -3  2  0 | 7.18 | 7.17 | 6 -5  0 | 15.10 | 15.11 | -7  5 -1 | 19.24 | 19.25 |
| -3  3  0 | 8.12 | 8.13 | -5  4 -1 | 15.37 | 15.38 | -3  1  2 | 19.50 | 19.49 |
| 3 -3  0 | 8.11 | 8.13 | 5 -4  1 | 15.36 | 15.38 | -3  2 -2 | 19.48 | 19.49 |
| -1  0 -1 | 9.39 | 9.42 | 4 -5  1 | 15.36 | 15.38 | 1 -3  2 | 19.50 | 19.49 |
| -1  0  1 | 9.44 | 9.42 | 6 -6  0 | 16.29 | 16.29 | 3 -2 -2 | 19.51 | 19.49 |
| 0 -1  1 | 9.44 | 9.42 | -5  5 -1 | 16.30 | 16.32 | -8  6  0 | 19.60 | 19.61 |
| 0  1 -1 | 9.43 | 9.42 | 5 -5 -1 | 16.29 | 16.32 | -7  6 -1 | 20.00 | 20.01 |
| 1 -1 -1 | 9.40 | 9.42 | 5 -5  1 | 16.30 | 16.32 | 7 -6  1 | 20.01 | 20.01 |
| 1 -1  1 | 9.42 | 9.42 | -7  5  0 | 16.96 | 16.97 | -4  2 -2 | 20.41 | 20.43 |
| 1  0 -1 | 9.42 | 9.42 | -6  4 -1 | 16.97 | 16.99 | -4  2  2 | 20.43 | 20.43 |
| -2  1 -1 | 10.15 | 10.17 | 5 -6  1 | 17.61 | 17.63 | 2 -4  2 | 20.43 | 20.43 |
| -2  1  1 | 10.19 | 10.17 | -7  6  0 | 17.80 | 17.82 | -8  7  0 | 20.52 | 20.54 |
| -1  2 -1 | 10.18 | 10.17 | 7 -6  0 | 17.81 | 17.82 | 8 -7  0 | 20.55 | 20.54 |
| 1 -2  1 | 10.19 | 10.17 | 0  0 -2 | 18.09 | 18.10 | -4  1  2 | 20.64 | 20.61 |
| 2 -1  1 | 10.17 | 10.17 | -1  0 -2 | 18.28 | 18.31 | -4  3 -2 | 20.59 | 20.61 |
| -2  2 -1 | 10.52 | 10.53 | 0 -1 -2 | 18.28 | 18.31 | -3  4 -2 | 20.63 | 20.61 |
| 2 -2 -1 | 10.51 | 10.53 | -1  0  2 | 18.30 | 18.31 | 3 -4  2 | 20.60 | 20.61 |
| 2 -2  1 | 10.53 | 10.53 | -1  1 -2 | 18.30 | 18.31 | -8  5  1 | 21.11 | 21.10 |
| 4 -4  0 | 10.82 | 10.84 | 0 -1  2 | 18.32 | 18.31 | -7  7 -1 | 21.07 | 21.10 |
| -3  2 -1 | 11.51 | 11.53 | 1 -1 -2 | 18.30 | 18.31 | 7 -7 -1 | 21.08 | 21.10 |
| -3  2  1 | 11.55 | 11.53 | 1 -1  2 | 18.29 | 18.31 | 7 -7  1 | 21.08 | 21.10 |
| 3 -2  1 | 11.52 | 11.53 | 1  0 -2 | 18.32 | 18.31 | -8  6 -1 | 21.61 | 21.63 |
| -5  3  0 | 11.83 | 11.82 | -6  6 -1 | 18.63 | 18.66 | 8 -6  1 | 21.62 | 21.63 |
| -3  3 -1 | 12.14 | 12.16 | 6 -6 -1 | 18.63 | 18.66 | -5  2  2 | 21.70 | 21.68 |
| 3 -3 -1 | 12.13 | 12.16 | 6 -6  1 | 18.63 | 18.66 | 3 -5  2 | 21.68 | 21.68 |
| 3 -3  1 | 12.15 | 12.16 | 1 -2 -2 | 18.68 | 18.71 | -5  4 -2 | 22.01 | 22.02 |
| -5  4  0 | 12.43 | 12.43 | -2  1  2 | 18.70 | 18.71 | 4 -5  2 | 22.00 | 22.02 |
| 5 -4  0 | 12.42 | 12.43 | -1 -1  2 | 18.72 | 18.71 | 5 -4  2 | 21.99 | 22.02 |
| -4  3 -1 | 13.30 | 13.32 | -1  2 -2 | 18.72 | 18.71 | -8  7 -1 | 22.45 | 22.48 |
| -3  4 -1 | 13.31 | 13.32 | 2 -1 -2 | 18.72 | 18.71 | 8 -7  1 | 22.46 | 22.48 |
| 4 -3  1 | 13.30 | 13.32 | -2  0  2 | 18.92 | 18.91 | 7 -8  1 | 22.45 | 22.48 |
| 5 -5  0 | 13.56 | 13.56 | 0 -2  2 | 18.91 | 18.91 | -9  6 -1 | 23.45 | 23.46 |
| -4  4 -1 | 14.11 | 14.12 | 2 -2 -2 | 18.90 | 18.91 | 9 -7  1 | 24.10 | 24.10 |
| 4 -4  1 | 14.11 | 14.12 | 2 -2  2 | 18.89 | 18.91 | 9 -9  0 | 24.47 | 24.55 |
| -6  4  0 | 14.37 | 14.36 | -8  5  0 | 19.05 | 19.03 |  |  |  |

Data were collected at 91(3) GPa and room temperature. Observed and calculated 2-theta angles are compared in the table with incident beam wavelength *λ* = 0.4066 Å. The second crystallite grain has 107 reflections with lattice parameters of *a* = 9.946(1) Å and *c* = 2.5852(2) Å.

**Supplementary Table 3.** Elemental analysis of hematite (Fe_2_O_3_), goethite (FeOOH), and the recovered sample of the OE-phase at ambient conditions.

| O:Fe ratio | Fe_2_O_3_ | The OE-phase | FeOOH |
| --- | --- | --- | --- |
| Measured values | 1.47(4) | 1.80(6) | 2.12(6) |
| Calibrated values | 1.5 (fixed) | 1.75(8) (corrected) | 2.0 (fixed) |

**Supplementary Table 4**. Atomic structure of the ferric OE-phase at 72(2) GPa and 300 K.

| O:Fe ratio | Fe_2_O_3+0.333_ | | | | Fe_2_O_3+0.5_ | | | | |
| --- | --- | --- | --- | --- | --- | --- | --- | --- | --- |
| Crystal system | Hexagonal | | | | Hexagonal | | | | |
| Space group | *P*6_3_ | | | | *P*6_3_ | | | | |
| *a* (Å) | 10.135(1) | | | | 10.135(1) | | | | |
| *b* (Å) | 10.135(1) | | | | 10.135(1) | | | | |
| *c* (Å) | 2.6418(5) | | | | 2.6418(5) | | | | |
| *α* (°) | 90 | | | | 90 | | | | |
| *β* (°) | 90 | | | | 90 | | | | |
| *γ* (°) | 120 | | | | 120 | | | | |
| *Z* | 6 | | | | 6 | | | | |
| *Atomic position* | *x* | *y* | *z* | *Occup.* | | *x* | *y* | *z* | *Occup.* |
| Fe1 | 0.383 | 0.537 | 0.250 | 1 | | 0.384 | 0.540 | 0.397 | 1 |
| Fe2 | 0.933 | 0.683 | 0.250 | 1 | | 0.931 | 0.680 | 0.337 | 1 |
| O1 | 0.354 | 0.404 | 0.750 | 1 | | 0.363 | 0.403 | 0.966 | 1 |
| O2 | 0.910 | 0.793 | 0.750 | 1 | | 0.087 | 0.223 | 0.342 | 1 |
| O3 | 0.458 | 0.725 | 0.750 | 1 | | 0.459 | 0.719 | 0.918 | 1 |
| O4 | 0.000 | 0.000 | 0.000 | 1 | | 0.930 | 0.930 | 0.000 | 0.5 |
